# The Population Structure of a Globe Artichoke Worldwide Collection, as Revealed by Molecular and Phenotypic Analyzes

**DOI:** 10.3389/fpls.2022.898740

**Published:** 2022-07-05

**Authors:** Domenico Rau, Giovanna Attene, Monica Rodriguez, Limbo Baghino, Anna Barbara Pisanu, Davide Sanna, Alberto Acquadro, Ezio Portis, Cinzia Comino

**Affiliations:** ^1^Dipartimento di Agraria, Sezione di Agronomia, Coltivazioni Erbacee e Genetica (SACEG), Università degli Studi di Sassari, Sassari, Italy; ^2^Agenzia AGRIS Sardegna (Servizio Ricerca sui Sistemi Colturali Erbacei, Settore Innovazione dei Modelli Gestionali e Studio Della Biodiversità Nelle Colture Intensive), Oristano, Italy; ^3^Dipartimento di Scienze Agrarie, Forestali ed Alimentari (DISAFA), Genetica Vegetale (Plant Genetics), Università degli Studi di Torino, Turin, Italy

**Keywords:** *Cynara cardunculus* var. *scolymus* L., divergent selection, germplasm collection, microsatellite, Q_ST_, quantitative traits, simple sequence repeats, UPOV traits

## Abstract

The knowledge of the organization of the domesticated gene pool of crop species is an essential requirement to understand crop evolution, to rationalize conservation programs, and to support practical decisions in plant breeding. Here, we integrate simple sequence repeat (SSR) analysis and phenotypic characterization to investigate a globe artichoke collection that comprises most of the varieties cultivated worldwide. We show that the cultivated gene pool of globe artichoke includes five distinct genetic groups associated with the major phenotypic typologies: Catanesi (which based on our analysis corresponds to Violetti di Provenza), Spinosi, Violetti di Toscana, Romaneschi, and Macau. We observed that 17 and 11% of the molecular and phenotypic variance, respectively, is between these groups, while within groups, strong linkage disequilibrium and heterozygote excess are evident. The divergence between groups for quantitative traits correlates with the average broad-sense heritability within the groups. The phenotypic divergence between groups for both qualitative and quantitative traits is strongly and positively correlated with SSR divergence (F_ST_) between groups. All this implies a low population size and strong bottleneck effects, and indicates a long history of clonal propagation and selection during the evolution of the domesticated gene pool of globe artichoke. Moreover, the comparison between molecular and phenotypic population structures suggests that harvest time, plant architecture (i.e., plant height, stem length), leaf spininess, head morphology (i.e., head shape, bract shape, spininess) together with the number of heads per plant were the main targets of selection during the evolution of the cultivated germplasm. We emphasize our findings in light of the potential exploitation of this collection for association mapping studies.

## Introduction

The study of the organization of the gene pool of crops is an essential prerequisite for both theoretical and practical reasons. This provides insight into the evolutionary history of a crop, strengthens our knowledge for the implementation of correct conservation strategies, and rationalizes operational choice in plant breeding.

For major crops, many studies on population structure are available, such as *Triticum aestivum* (Würschum et al., 2013), *Oryza sativa* (Jin et al., 2010), *Zea mays* (Vigouroux et al., 2008) *Hordeum vulgare* (Malysheva-Otto et al., 2006; Tanto Hadado et al., 2010; Rodriguez et al., 2012), *Lycopersicon esculentum* (Aflitos et al., 2014; Rodriguez et al., 2020), *Solanum melongena* (Cericola et al., 2013, 2014; Portis et al., 2015), *Phaseolus spp.* (Angioi et al., 2010; Bitocchi et al., 2013; Rodriguez et al., 2013), and *Glycine max* (Bandillo et al., 2015). However, for other minor crops, such as *Cynara cardunculus* var. *scolymus* L., the cultivated globe artichoke, information remains limited.

Globe artichoke (*2n* = *2x* = 34) is a perennial open-pollinated crop (De Vos, 1992; Pecaut, 1993) that is probably native to the Mediterranean basin (Sonnante et al., 2007; Gatto et al., 2013) where the cultivation of this crop is also particularly relevant. Based on data from the Food and Agriculture Organization Corporate Statistical Database^1^ (FAOSTAT, 2020), the total world artichoke production in 2020 was 1,516,955 tons. In total, three circum-Mediterranean countries, namely Italy (367,080 tons), followed by Egypt (308,844 tons), and Spain (196,970 tons), were the most important world producers, accounting for 57.5% of the global production.

The edible portion of the artichoke plant is the head, or capitulum, which is formed as a composite inflorescence that is harvested before the flowers bloom. Furthermore, globe artichoke is considered a functional food and a source of nutraceutical ingredients, such as bioactive phenolic compounds, inulin, fiber, and minerals; in addition, artichoke leaf extracts have long been used in folk medicine, particularly for liver complaints (Comino et al., 2007, 2009; Lattanzio et al., 2009; Moglia et al., 2009, 2016; Menin et al., 2010, 2012; Eljounaidi et al., 2014, 2015).

Many different clonal varietal groups best adapted to different local environments have been described in this species. Cultivated germplasm has traditionally been classified into four main groups based on the capitulum traits (Dellacecca et al., 1976; Porceddu et al., 1976; Vanella et al., 1981): (1) The “Spinosi” group has long spines on the bracts and leaves; (2) “Violetti” has medium-sized, violet-colored, and less spiny heads; (3) “Romaneschi” has spherical or subspherical non-spiny heads; and (4) “Catanesi” has relatively small, elongated, and non-spiny heads. Elia and Miccolis (1996) conducted a phenotypic characterization in a common garden experiment. They conducted a multivariate analysis considering eight quantitative traits across 104 accessions, and they distinguished five main morphophenological clusters. These were early; medium-early; late with small heads; late-violet; and late with large heads. Furthermore, Lanteri et al. (2004) studied a wide collection of cultivated globe artichoke using amplified fragment length polymorphism (AFLP) markers and found concordances between molecular and the proposed phenotypic classifications. Indeed, the performed multivariate analysis showed four main clusters that corresponded to the phenotypical typologies of cultivated globe artichoke: “Catanesi” (together with “Violet de Provence”), “Romaneschi,” “Violetti di Toscana,” and “Spinosi.”

Later, by analyzing leafy cardoon and globe artichoke accessions using SSR and inter-SSR markers, Pagnotta et al. (2017) observed that “Catanesi” resulted well separated from the other accessions, and the “Spinosi” types formed a well-defined group together with some “Romaneschi” and “Violetti” types. The “Romaneschi” types showed low uniformity, as they were distributed into several genetic subgroups. Thus, except for the “Catanesi” group, the association between molecular groups and phenotypic types was not clear. Moreover, as noted by Pagnotta et al. (2017), the accessions clustered not only based on their typology but also based on the gene bank of origin. They argued that the environmental conditions of the different field gene banks or the methods used to conserve plant materials might have affected the germplasm characteristics.

More recently, Pavan et al. (2018) used genotyping by sequencing to analyze samples of globe artichoke, cultivated cardoon (*C. cardunculus* var. *altilis*), and wild cardoon (*C. cardunculus* var. *sylvestris*), and showed a clear-cut genetic separation between globe artichoke and the cultivated and wild cardoon. When the analysis was repeated assuming three genetic groups, the authors observed the separation of “Catanesi” from all of the other accessions and that the group of green-headed landraces typical of the Apulia region, Italy (Green Apulian), also formed a well-defined group. Furthermore, similarly to Pagnotta et al. (2017) and Pavan et al. (2018) observed that “Romaneschi” were attributed to a variable group that consisted of admixed individuals, which suggested that they should not be considered a genetically uniform varietal typology.

To the best of our knowledge, no studies are available that compare the molecular and phenotypic population structures for the same collection of globe artichokes. Thus, in this study, we characterized a germplasm collection comprising 110 of the most important globe artichoke varieties cultivated worldwide by conducting a deep phenotypic characterization in a common garden and under field conditions. A total of thirty-five quantitative phenological, morphological, and yield traits and 23 qualitative descriptors from the International Union for the Protection of New Varieties of Plants (UPOV) were considered to describe the plants in the stages of the first, secondary, and tertiary heads. Thus, the phenotypic data were compared with the results of SSR analysis, to investigate the relationships between molecular and phenotypic variability.

This study advances our understanding of the evolution of globe artichoke, strengthens the knowledge of the organization of its gene pool, and can help in rationalizing the operational choices when applying association mapping and defining breeding strategies in this species.

## Materials and Methods

### Plant Material

A total of 110 globe artichoke accessions were analyzed using both SSR and quantitative traits, as listed in Supplementary Table 1. All of these came from the living artichoke collection maintained by AGRIS at the experimental farm located at Oristano (Sardinia, Italy). The collection comprises most of the globe artichoke varieties cultivated worldwide, with the accessions subdivided into seven main typologies based on their different morphophenotypic traits (primarily related to head characteristics). These typologies are named as “Catanesi” (CAT), “Violetti di Provenza” (VPR), “Violetti di Toscana” (VTO), “Romaneschi” (ROM), “Macau” (MAC), “Spinosi” (SPI), and “Green et al.” (GEA), as reported by Comino et al. (2016).

Nine clones of “Spinoso sardo” selected by AGRIS based on morphological and production traits were also analyzed (Supplementary Table 1).

### Simple Sequence Repeat Assay

Total genomic DNA was extracted from young leaves (three plants per accession) and characterized using 26 genomic-SSR (gSSR) markers targeting loci distributed through all of the 17 chromosomes of globe artichoke (Supplementary Table 2A). The markers were selected according to their good resolution power and reliability (Scaglione et al., 2009, 2016; Portis et al., 2012, 2016).

Chloroplast-SSR (SR) variation was also investigated using 10 primer pairs designed by Weising and Gardner (1999) and 23 designed by Chung and Staub (2003) (Supplementary Table 2B); the markers have been already used to study the population structure of wild cardoon, the progenitor of globe artichoke (Rau et al., 2016), and other plants (see, e.g., Angioi et al., 2009, 2010; Desiderio et al., 2013).

### Individual-Based Clustering

To infer the population structure of the globe artichoke collection using multilocus genetic profiles, we first used the model-based (Bayesian) clustering algorithm implemented in the Structure software (Pritchard et al., 2000; Falush et al., 2003). The data were analyzed by setting the admixture model and the correlated allele frequencies options. All runs were carried out with 50,000 burn-ins and 100,000 iterations (Falush et al., 2003). Phenotypic typology was not used as prior information to assist clustering. A total of twenty independent runs were carried out for each number of assumed ancestral populations (K). A range of K from 1 to 10 was explored (Evanno et al., 2005). The K value that best explained the data was determined using the *ad hoc* statistics (ΔK; Evanno et al., 2005) with the CLUMPAK software (Kopelman et al., 2015).

To complement the population structure analysis, discriminant analysis of principal components (DAPC) was used as a multivariate unsupervised method implemented in the R package *adegenet* (Jombart, 2008; Jombart et al., 2010). DAPC divides genetic variation into between- and within-group components, to maximize the former, and builds linear combinations of alleles (linear discriminants) that best separate the groups. The *find*.*clusters* function was used to detect the number of clusters in the collection adopting the K-means clustering method and determining the most likely number of subpopulations based on the Bayesian Information Criterion (BIC). The number of principal components to be retained was determined using the cross-validation function *Xval*.*dapc*, based on the root mean square error.

### Variation Among Genetic Groups

The total SSR genetic variance (σ^2^_*T*_) was partitioned into two components: among-population (σ^2^_a_) and within-population (σ^2^_b_). The divergence between populations was quantified as F_ST_ = σ^2^_a_/σ^2^_*T*_, where σ^2^_*T*_ = σ^2^_a_ + σ^2^_b_. The partition of molecular variance was accomplished by the AMOVA implemented in Arlequin v. 3.1.5.2 (Excoffier and Lischer, 2010). The level of group divergence was also quantified by calculating the R_ST_. While F_ST_ is based on the infinite allele model (IAM), R_ST_ is based on the stepwise mutation model (SMM), i.e., it also considers the variance of SSR allele size (Gaggiotti et al., 1999). The F_ST_ and R_ST_ statistics have the same expectations when group divergence is caused only by drift, whereas R_ST_ is larger than F_ST_ if stepwise-like mutations are also contributing to divergence; thus, comparing the F_ST_ and R_ST_ values can provide insights into the relative contribution of drift and mutation to group divergence (Hardy et al., 2003). This comparison can be done by testing whether the observed R_ST_ is significantly larger than its value after randomizing allele sizes (pR_ST_; Hardy et al., 2003). If the R_ST_ is not significantly different from pR_ST,_ it is possible to conclude that R_ST_ = F_ST_ (Hardy et al., 2003). The *p*-values were obtained after 999 random permutations using the SPAGeDI software (Hardy and Vekemans, 2002).

### Diversity and Linkage Disequilibrium Within Genetic Groups

The number of alleles per locus (n_a_), the observed heterozygosity (H_O_), and the gene diversity (H_E_, Nei, 1978) were estimated for each genetic group identified by the Structure and DAPC analyzes. When referring to the qualitative traits variation, to avoid confusion with SSR, we indicate the number of classes per trait as n_*c*_ and the gene diversity of Nei (1978) as I_Nei_ (instead of H_E_). To determine the coefficient of inbreeding (F_IS_), total variance (σ^2^_*T*_) was partitioned into three components: among populations (σ^2^_a_), among individuals within-populations (σ^2^_b_), and within individuals (σ^2^_*c*_). The total F_IS_ was calculated as σ^2^_b_/(σ^2^_b_ + σ^2^_*c*_). Group-specific F_IS_ was also calculated. This coefficient compares the expected and observed heterozygosities and the expected value under the Hardy-Weinberg equilibrium is 0. Thus, F_IS_ > 0 indicates heterozygote deficit compared to the Hardy-Weinberg equilibrium (as a consequence of inbreeding), while F_IS_ < 0 indicates heterozygote excess (as in clonally propagated crops or when selection in favor of heterozygotes is applied).

The BOTTLENECK ver. 1.2 software (Piry et al., 1999) was used to determine whether the globe artichoke groups experienced recent founder events or population bottlenecks. In this case, both a reduction of the number of alleles (n_a_) and of expected heterozygosity (H_E_) is expected, with the former that reduces faster than the latter (Nei, 1975). Given n_a_ and the sample size, the program BOTTLENECK computes, for each sample and for each locus, the distribution of the heterozygosity expected under the assumption of mutation-drift equilibrium (H_*EQ*_). As n_a_ reduces faster than H_E_, it is expected that, on average, after a bottleneck H_E_ > H_*EQ.*_ (Cornuet and Luikart, 1996). The number and percentage of pairs of SSR loci in linkage disequilibrium (LD; *p* < 0.05) were calculated using the Arlequin software (Excoffier and Lischer, 2010).

### Phenotypic Characterization and Statistical Analysis

The collection was characterized in the AGRIS experimental field (Azienda Palloni, Oristano, Sardinia, Italy). Individual plants were obtained by vegetative propagation using “*ovoli*” in July. The plants were managed following standard agronomic practices for the crop, both for mineral nutrition and water supply.

Phenotypic data were recorded under field conditions, measuring three plants for each variety (single plant = replicate) in a completely randomized design. A total of twenty-one traits were recorded at the beginning of the first head harvest (Supplementary Table 3A). Among these, 11 traits were also recorded for the secondary and tertiary heads (Supplementary Table 3B). The traits “Total number of heads” and “Total weight of heads” were measured for secondary and tertiary heads. The collection was also characterized by recording 23 qualitative phenotypic descriptors defined by UPOV (Supplementary Table 3C).

The total phenotypic variance (σ^2^_*T*_) was decomposed into two components, within SSR groups (σ^2^_W_) and between SSR groups (σ^2^_B_), applying Nested Analysis of Variance (NANOVA). For each trait, the variance associated with each component was estimated considering a model with groups, varieties within groups, and replicates as random factors and applying the restricted maximum likelihood (REML) method implemented in the JMP software (ver. 10.0.0, SAS, Institute, NC, United States).

Variance components were first used to calculate the heritability of traits. In globe artichoke, cross-fertilization is promoted by protandry (Mauromicale and Ierna, 2000), and when the progenies are tested, they show wide segregation of morphological and production traits (Basnitzki and Zohary, 1987). Thus, high heterozygosity was expected *a priori*. As the globe artichoke varieties considered in this study were obtained by vegetative propagation (clonally), it is assumed that the three replicates of each variety have the same genotype. This means that genetic differences among varieties, both within groups (σ^2^_W_) and between groups (σ^2^_B_), can be partially due to maternal and non-additive effects (dominance and epistasis), rather than to additive effects. Thus, the sum σ^2^_W_ + σ^2^_B_ is equal to the total genetic variance (σ^2^_*G*_) and the ratio between genetic variance (σ^2^_*G*_) and total variance (σ^2^_*T*_) represents the broad-sense heritability (h^2^_B_); i.e., h^2^_B_ = σ^2^_*G*_/σ^2^_*T*_, where σ^2^_*T*_ = σ^2^_W_ + σ^2^_B_ + σ^2^_E_, with σ^2^_E_ representing the environmental variance.

To explore the pattern of phenotypic divergence between genetic groups of globe artichoke varieties, variance components were used to calculate the degree of differentiation for quantitative traits between groups by applying the formula Q_ST_ = σ^2^_B_/(σ^2^_B_ + 2σ^2^_W_) (Spitze, 1993; Whitlock, 1999; Leinonen et al., 2008). The Q_ST_ statistic was introduced by Spitze (1993) as an analog to F_ST_ for molecular markers. This equation assumes that F_IS_ = 0 and that σ^2^_B_ is the *additive* genetic variance due to differences between populations, and σ^2^_W_ is the mean *additive* genetic variance within populations. Successively, Bonnin et al. (1996) derived a generalized relation for any possible value of F_IS_, for which Q_ST(f)_ = (1 + F_IS_)σ^2^_*B/*_[(1 + F_IS_)σ^2^_B_ + 2σ^2^_W_]. To calculate the Q_ST_s, we also applied this formula by entering the estimated total F_IS_ (see previous paragraph) and assuming that the F_IS_ of a quantitative trait locus is the same as the F_IS_ of the neutral markers. We presented two Q_ST_ values calculated as in Spitze (1993) and Bonnin et al. (1996). In our case, σ^2^_B_ and σ^2^_W_ are components of the total genetic variance, as they can include both non-additive and maternal effects. To mark this difference compared to Spitze’s Q_ST_ and Bonnin’s Q_ST(f)_, we used the notation Q_ST_B_ and Q_ST(f)_B_, where the subscript “B” means “Broad.”

The qualitative descriptors of the UPOV were statistically treated as molecular markers, considering each trait as a single SSR marker and the states of the trait as different alleles. Thus, for each group, statistics of genetic diversity (n_a_, H_E_) and divergence (F_ST_*qlt*_) were calculated as for the SSR markers (Tanto Hadado et al., 2009). The correlations between the three divergence measures (i.e., F_ST_, Q_ST_B_, F_ST_qlt_) were determined. The values of F_ST_*qlt*_ were compared with the F_ST_ values obtained for SSRs to flag qualitative traits putatively under selection, using the Arlequin software v. 3.5.1.2 (see below).

The levels of molecular and quantitative variations were compared following the rationale of the F_ST_ – Q_ST_ approach (Spitze, 1993; Whitlock, 1999; Leinonen et al., 2008). This is based on the relative magnitude of the F_ST_ and Q_ST_ and allows inferences to be made about the relevance of selection in the evolution of a specific quantitative trait (see “Discussion” section). To exclude possible selection effects on F_ST_ values calculated for the SSR loci, the (putatively) neutral “benchmark” F_ST_ was calculated by conducting the outlier tests as implemented in Arelquin v. 3.5.1.2 (100,000 reps). This test is similar to that implemented in FDIST computer program (Beaumont and Nichols, 1996). We ran simulations reiteratively, eliminating after each simulation the markers that showed the signature of divergent selection (*p* < 0.05). After obtaining a dataset without markers with signatures of divergent selection, the average F_ST_ across loci was assumed as the putatively neutral benchmark. The confidence intervals (C.I.) of the putatively neutral F_ST_ were determined by bootstrapping over loci (100,000 replicates).

## Results

### Population Structure Based on Genomic SSRs

All SSR markers tested were polymorphic (Minimum Allele Frequency, MAF, > 1%). A total of 152 alleles were observed: two to 13 alleles per locus (n_e_) were detected, giving a mean of 5.85. Gene diversity (H_E_) per locus varied from 0.231 to 0.822 (Supplementary Table 2A). When the 26 SSRs were combined, each accession showed a unique multilocus profile.

**FIGURE 1:**
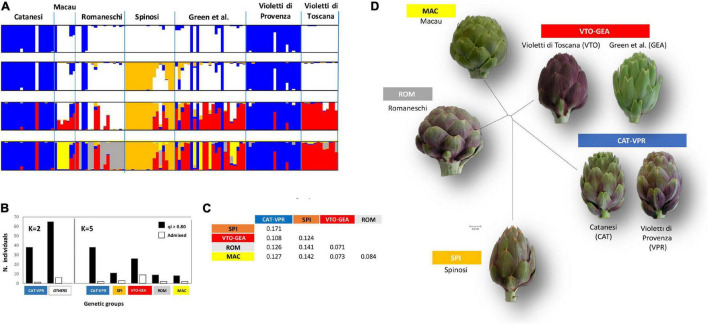
Structure analysis. **(A)** Individual to population assignment. Individuals are ordered and grouped according to *a priori* knowledge of phenotypic typology. **(B)** Distribution between groups of individuals assigned (q_*i*_ > 0.80) or admixed (*q* < 0.20) at *K* = 2 and *K* = 5. **(C)** Net-nucleotide distances between pairs of genetic groups (*K* = 5). **(D)** Neighbor-joining (N-J) tree depicting distances between groups (*K* = 5); the N-J tree was drawn by Structure software based on the pairwise net-nucleotide distances among the five detected groups.

Based on Evanno’s ΔK statistics, the highest hierarchical level of the population structure was *K* = 2 (Supplementary Figure 1). However, a secondary peak at *K* = 5 was also evident, which indicated substructures (Supplementary Figure 1). The genetic subdivisions resembled phenotypic typologies quite well (Figure 1A). Indeed, at *K* = 2, most of the Catanesi and Violetti di Provenza types clustered together and were clearly separated from the remaining accessions; most of the individuals (> 95%) were assigned to their group with q_*i*_ > 0.80 (Figure 1B). The two additional clustering steps first separated most Spinosi (*K* = 3) and then a group that included all the Violetti di Toscana and most green-headed types (*K* = 4; Figure 1A). Finally, at *K* = 5, most of the Romaneschi were split from Macau. At *K* = 5 Catanesi and Violetti di Provenza still clustered together, while Violetti di Toscana grouped with many Green-headed accessions (Figure 1A). This remained true for K up to 10 (not shown). The phenotypic typology “Green et al.” was not genetically well-defined. Indeed, it comprised varieties assigned to different genetic groups that were typical of Violetti di Toscana, Macau, and Catanesi-Violetti di Provenza (Figure 1A).

Based on these data, from here on, we adopted the label CAT-VPR to designate the nuclear SSR genetic group related to the Catanesi-Violetti di Provenza phenotypic typology; similarly, the labels SPI, VTO-GEA, ROM, and MAC indicate the genetic groups associated with the phenotypic typologies Spinosi, Violetti di Toscana-Green et al., Romaneschi, and Macau, respectively. Overall, at *K* = 5, most of the accessions were assigned to their group with high probability (q_*i*_ > 0 80; Figure 1B). Overall, there was a strong association between phenotypic typologies and genetic classifications (χ^2^ test: *p* < 10^–4^). The highest frequency (∼30%) of admixed individuals (q_*i*_ < 0.80) was observed within the VTO-GEA group.

CAT-VPR and SPI showed the highest net-nucleotide distance (0.171), while the lowest distances were observed between MAC and ROM (0.084), VTO-GEA and MAC (0.073), and VTO-GEA and ROM (0.071) (Figure 1C). According to AMOVA, the average genetic divergence (F_ST_) between these five groups was moderate, being 0.170 (Table 1) while putatively neutral F_ST_ was slightly lower (0.134).

**TABLE 1:** Genetic divergence (F_ST_, R_ST_) among the five groups of varieties of cultivated globe artichoke as determined by AMOVA over loci considering two sources of variation: among populations and among individuals within populations.

Fixation index	Value	*P*-value	Median	Confidence interval		
				95%	99%	99.9%
F_ST_	0.170	<10^–5^	0.169	0.137–0.203	0.125–0.216	0.111–0.230
F_ST (PN)_	0.134	<10^–5^	0.134	0.111–0.156	0.101–0.166	0.089–0.175
R_ST_	0.148	<10^–5^	0.148	0.098–0.202	0.083–0.221	0.066–0.245
pR_ST_	0.179	0.364		0.119–0.239		

*p-value: for observed F_ST_ and R_ST,_ p (rand. value ≥ obs. value); for pR_ST_, p (2-sided test, obs. value < > simulated value randomizing alleles across individuals).*

*Median, Confidence interval, obtained by bootstrapping over loci.*

*F_ST_, from locus-by-locus AMOVA, with the within-individual level with all of the SSRs.*

*F_ST (PN)_, from locus-by-locus AMOVA, with the within-individual level and after excluding four loci significant (p < 0.05) at the F_ST_ outlier test for divergent selection; PN, putatively neutral.*

*R_ST_, from locus-by-locus AMOVA, with the within-individual level with all of the SSRs.*

*pR_ST_ (and its confidence interval) obtained by shuffling alleles across individuals.*

According to R_ST_, the average divergence among the genetic groups was 0.148 (Table 1). Furthermore, the R_ST_ and F_ST_ values were not statistically different (Table 1) because the observed R_ST_ (0.148) and the R_ST_ calculated by randomizing allele size were not different (pR_ST_ = 0.179; *p* = 0.364; Table 1). Therefore, in this study, stepwise-like mutations are not contributing to group divergence. The neighbor-joining tree emphasizes the proximity between ROM, MAC, and VTO-GEA, and their distance from CAT-VPR and SPI (Figure 1D).

**FIGURE 2:**
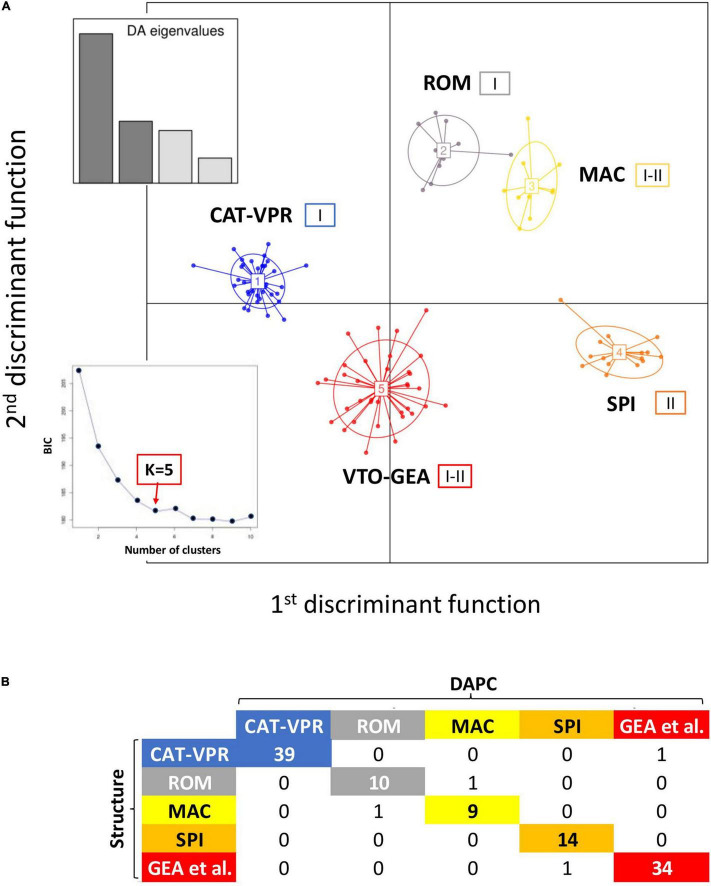
Discriminant analysis of the principal components (DAPC). **(A)** Five groups of cultivated globe artichoke varieties as depicted by the first two discriminant functions. Insets, left: eigenvalues for the first four discriminant functions. The small square embedded in the lower left part of the figure represents the BIC values as a function of the numbers of assumed genetic groups. Roman numbers (I and II) indicate the chloroplast alleles observed within each nuclear SSR group. **(B)** Correspondence between the Structure and DAPC classifications.

When applying the DAPC analysis and the function *find.cluster* with the K-mean clustering method, the BIC values plot showed a knee when the number of groups was five, although the minimum BIC value was observed for *K* = 9 (Figure 2A). Thus, based on the BIC, five was a parsimonious number of clusters for the modeling of our data. DAPC was carried out considering the five groups detected by the K-means clustering method. Four linear discriminants were maintained for the analysis (Figure 2A). The first two linear discriminants were sufficient to identify five groups that essentially corresponded to those previously identified by Structure (Figure 2A). At opposite extremes of the first linear discriminant, there were CAT-VPR and SPI; the second linear discriminant mainly separated VTO-GEA from both ROM and MAC. The third and the fourth linear discriminants refined the relationships among the groups (Supplementary Figure 2). For further details on the classification of the individual accessions provided by DAPC see Supplementary Figure 3.

The correspondence between the Structure and DAPC classifications is reported in Figure 2B. Only four varieties were classified into different groups by the two methods (Figure 2B). These were Testa di Ferro, Gross Camus, Spinoso di Gonnos, and Spinoso di Gela that, based on Structure, were all admixed, with the coefficient of membership (q_*i*_) to the prevalent genetic groups between 0.489 and 0.644. In two of the four cases, the “mismatches” between the two clustering methods involved varieties that were moved between closely related groups. Indeed, Spinoso di Gonnos was assigned to ROM by Structure, while it was assigned to MAC by DAPC; on the contrary, Testa di Ferro was assigned to MAC by Structure, while it was assigned to ROM by DAPC.

Therefore, both the model-based (Structure) and the unsupervised (DAPC) methods agreed when identifying five groups.

### Genomic SSR Polymorphism Within Groups

There was a significant difference between the groups for the number of alleles per locus (n_a_) (Wilcoxon’s test: χ^2^ = 25.381; d.f. = 4; *p* = 0.00004; Table 2) with VTO-GEA that showed the highest value. The groups also showed significantly different levels of gene diversity (H_E_) (Wilcoxon’s non-parametric test: χ^2^ = 18.69, d.f. = 4; *p* = 0.0009). The VTO-GEA group showed the highest H_E_ and was significantly more diverse than SPI and CAT-VPR, which showed the lowest H_E_ values; ROM and MAC were in intermediate positions (Table 2).

**TABLE 2:** Descriptive genetic statistics for the five SSR groups identified within the gene pool of the cultivated globe artichoke according to the Structure and DAPC analyzes.

Molecular group	N° of alleles per locus (n_a_)	N° polymorphic SSR loci	Polymorphism (%)	Gene diversity (H_E_)	Inbreeding (F_IS_)^1^	N° loci deviating HW equilibrium	*P* (rand. F_IS_ ≥ Obs.)	*LD*	
								N° of pairs (*p* < 0.05)	(%)
CAT-VPR	3.1 b	25	96.2	0.440 b	−0.617 b	17 (23)	1.000	168	56.0
ROM	3.3 b	26	100.0	0.570 ab	−0.440 b	12 (23)	0.999	117	36.0
MAC	3.3 b	24	92.3	0.516 ab	−0.051 a	0 (15)	0.694	86	31.2
SPI	2.8 b	24	92.3	0.438 b	−0.445 b	9 (22)	1.000	129	46.7
VTO-GEA	5.1 a	26	100.0	0.606 a	−0.122 a	6 (18)	0.999	116	35.7
**Mean**	**3.5**	**25.0**	**96.2**	**0.514**	−**0.317**	**11.6 (17.6)**	**0.939**	**123**	**41.1**

*^1^Determined by single-locus AMOVA with the within-individual level.*

*The number of loci deviating from the H-W equilibrium is given for p < 0.001 (outside parentheses) and for p < 0.05 (within parentheses).*

AMOVA analysis performed considering the hierarchical level “within-individual” showed a global negative and significant coefficient of inbreeding, F_IS_ (−0.317, *p* < 10^–5^; Table 2; C.I._99%_ from −0.408 to −0.153) that indicated an excess of heterozygotes within the five genetic groups. In all cases except MAC, the group-specific F_IS_ were negative and statistically significant (*p* < 10^–5^). The F_IS_ value varied significantly between groups (Wilcoxon’s non-parametric test: χ^2^ = 34.61; d.f. = 4; *p* = 5.58 × 10^–7^). CAT-VPR, SPI, and ROM showed stronger excess of heterozygotes than MAC and VTO-GEA (Table 2). Bottleneck analysis confirmed frequent significant excess of heterozygosity compared to the expectation under mutation-drift equilibrium. This was more evident when a pure IAM model was considered when the frequency of loci evolving under SMM was < 70%, and for CAT-VPR, ROM, and SPI (Supplementary Table 4).

In general, the level of LD within the groups was relatively high, ranging from 35.7% (VTO-GEA) to 56.0% (CAT-VPR), with an average of 41.1% of pairs of loci in LD (*p* < 0.05). The three groups with the highest LD values were also those with the lowest F_IS_ (i.e., CAT-VPR, SPI, ROM) (Table 2).

### Chloroplast SSR Analysis

Universal cpSSR primers revealed low levels of cytoplasmic variability. Indeed, of the 27 assayed loci, only one (cpSSR-7) was polymorphic, with two alleles hereafter named “I” and “II.” The “I” allele was the most frequent (0.67) (Supplementary Figure 4). However, the chloroplast and nuclear patterns of variation were correlated (Supplementary Figure 4; χ^2^ test: *p* < 0.01). The two most divergent nuclear SSR groups (CAT-VPR, SPI) also show alternative cpSSR alleles: CAT-VPR was monomorphic for allele “I,” while SPI was monomorphic for allele “II” (Figure 2).

### Patterns of Phenotypic Variation Within and Between the SSR-Derived Groups

Table 3 reports the results of the NANOVA for the 21 quantitative traits measured at the harvest of the first head. Within the SSR groups, the varieties showed different means for all of the quantitative traits (*p* < 10^–5^). The broad-sense heritability (h^2^_B_) within groups varied widely depending on the traits considered, from 0.48 (stem diameter) to 0.98 (harvest time) (Table 3), with an average of 0.65. The five SSR-derived groups showed different mean h^2^_B_ across traits. Indeed, these ranged from 0.56 (CAT-VPR) to 0.77 (VTO-GEA), with ROM (0.60), MAC (0.63), and SPI (0.70) in intermediate positions. These differences between groups were statistically significant based on ANOVA (*p* < 0.01), while the Tukey-Kramer HD test separated CAT-VPR from the other four groups (*p* < 0.05).

**TABLE 3:** Nested Analysis of Variance (NANOVA) to test the significance of the differences among the varieties within the SSR groups and of the differences between the SSR groups for the 21 traits measured at the stage of the harvest of the first head.

Trait	Variance						Average heritability within groups (h^2^_B_)		
	Within SSR groups			Between SSR groups				Divergence between groups	
	SS	F	*P*	SS	F	*P*		(Q_ST_B_)^1^	[Q_ST(f)_B_]^2^
Harvest time	243,597.7	214.0	**** *	151,634.0	15.53	**** *	0.977	**0.200**	0.147
Plant height	94,616.0	21.1	**** *	74,019.9	19.45	**** *	0.748	**0.279**	**0.210**
Polar length of head	410.6	7.3	**** *	66.9	4.03	**	0.648	0.102	0.073
Equatorial length of head	242.5	4.9	**** *	29.4	2.98	*	0.616	0.112	0.080
Ratio polar/equatorial length of head	7.1	19.3	**** *	1.9	6.77	**** *	0.812	**0.223**	**0.164**
Weight of head	1,042,590.3	5.0	**** *	45,672.9	1.08	n.s.	0.582	0.000	0.000
Length of stem	18,954.3	10.5	**** *	3,701.6	4.84	**	0.637	**0.183**	0.133
Diameter of stem	28.0	5.1	**** *	1.2	1.09	n.s.	0.476	0.011	0.007
Weight of stem	1,549,831.0	8.5	**** *	170,423.4	2.72	*	0.598	0.091	0.065
Weight of head + stem	4,199,756.2	6.2	**** *	329,774.6	1.94	n.s.	0.582	0.059	0.041
N° of leaves on stem	306.4	8.2	**** *	22.3	1.80	n.s.	0.518	0.010	0.007
Weight of the leaves on stem	3,508,214.1	8.7	**** *	493,150.1	3.48	*	0.637	0.063	0.044
Weight of head + stem + leaves	10,850,343.3	7.5	**** *	1,178,972.7	2.69	*	0.685	0.066	0.047
Bract height	775.5	4.84	**** *	86.2	2.61	*	0.540	0.079	0.055
Bract width	8,331.6	14.72	**** *	2,614.4	7.38	****	0.765	**0.163**	0.118
Bract height/width	0.9	11.84	**** *	0.3	8.10	****	0.787	0.151	0.109
Bract thickness	169.0	7.61	**** *	25.9	3.60	**	0.619	0.114	0.081
Ten-bracts weight	28,020.8	8.52	**** *	3,283.5	2.75	*	0.632	0.070	0.049
Receptacle height	1,588.4	4.24	**** *	116.6	1.72	n.s.	0.519	0.014	0.009
Receptacle width	19,935.1	6.96	**** *	3,704.2	4.37	**	0.674	0.113	0.081
Receptacle height/width	0.7	3.61	**** *	0.2	5.58	***	0.495	0.120	0.086

*p < 0.05; **p < 0.01; ***p < 0.001; ****p < 0.0001; *****p < 10^–5^; n.s., not significant (Tukey-Kramer multiple comparison tests).

*Gray shading, traits for which the SSR groups have different means (p < 0.05; Tukey-Kramer multiple comparison tests).*

*For each trait, the value of the Q_ST_B_ is reported; Q_ST_B_ values are reported assuming F_IS_ = 0.00, or for the putatively neutral F_IS_ = −0.315 estimated from the SSR data.*

*The results of the F_ST_ – Q_ST_ comparisons are reported: underlined Q_ST_ values exceeded the upper 95% limit of the putatively neutral F_ST_ among the SSR groups; Q_ST_ values in bold exceeded the upper 99.5% limit; Q_ST_ values in bold and underlined exceeded the upper 99.9% limit.*

Based on ANOVA and Tukey-Kramer HD multiple comparison test, 10 out of 21 quantitative traits differed between the five genetic groups (with *p*-values from < 0.01 to < 10^–5^; Table 3). The strongest group divergence was seen for plant height (Q_ST_B_ = 0.279), polar/equatorial head-length ratio (Q_ST_B_ = 0.223), harvest time (Q_*ST_B*_ = 0.200), and length of stem (Q_ST_B_ = 0.183) (Table 3). Among the traits that defined the size and shape of the bracts and the receptacle, the width of the bract and bract height/length ratio was the most diverse among the groups (Q_ST_B_ = 0.163, 0.151, respectively; *p* < 10^–4^ in both cases; Table 3). The genetic groups showed low divergence for weight of first head (Q_ST_B_ = 0.00), diameter of stem (Q_ST_B_ = 0.011), number of leaves on stem (Q_ST_B_ = 0.010), and receptacle height (Q_ST_B_ = 0.014).

When considering the stages of the harvest of the secondary and tertiary heads, these globe artichoke varieties showed statistically different means (*p* < 10^–5^) for all the traits examined (Table 4). Again, the SSR-derived groups were strongly divergent for head shape (polar/equatorial head-length ratio, Q_ST_B_ = 0.26 and 0.28, at the two considered stages, respectively) and length of the stem (Q_ST_B_ = 0.28, 0.22, respectively), and to a lesser extent, for harvest time (Q_ST_B_ = 0.17, 0.16, respectively). Furthermore, this analysis showed differences in yield performances between the genetic groups, as Q_ST_B_ were 0.17 and 0.12 for the secondary and tertiary heads, respectively. Among the yield components, Q_ST_B_ tended to be higher for the average number of heads (0.13, 0.14, respectively) than for the average weight of the heads (0.11, 0.02, respectively). As expected, when Q_ST_B_ was calculated considering the observed average negative F_IS_, the Q_ST_ values were reduced (Tables 3, 4).

**TABLE 4:** Nested Analysis of Variance (NANOVA) for the 11 traits measured at the stage of secondary and tertiary heads.

	Secondary heads				Tertiary heads				Average		
Trait	h^2^_B_	Q_ST_B_	Q_ST(f)_B_	*p*	h^2^_B_	Q_ST_B_	Q_ST(f)_B_	*p*	h^2^_B_	Q_ST_B_	Q_ST(f)_B_
Average harvest time	0.89	**0.17**	0.12	**** *	0.89	0.16	0.12	**** *	0.89	0.16	0.12
Average polar length of head	0.79	0.16	0.11	***	0.88	0.12	0.08	***	0.83	0.14	0.10
Average equatorial length of head	0.61	0.15	0.11	***	0.65	0.03	0.02	n.s.	0.63	0.09	0.07
Average ratio polar/equatorial length	0.74	**0.26**	**0.19**	****	0.73	**0.28**	**0.21**	**** *	0.74	**0.27**	**0.20**
Average number of heads	0.34	0.13	0.09	****	0.51	0.14	0.10	**** *	0.42	0.13	0.10
Average weight of heads	0.65	0.11	0.08	***	0.71	0.02	0.02	n.s.	0.68	0.07	0.05
Total weight of heads	0.53	**0.17**	0.13	**** *	0.58	0.12	0.09	**** *	0.55	0.15	0.11
Average length of the stem	0.65	**0.28**	**0.20**	***	0.66	**0.22**	0.16	****	0.65	**0.25**	**0.18**
Average diamter of the stem	0.44	−0.01	−0.01	n.s.	0.63	0.05	0.03	n.s.	0.53	0.02	0.01
Average weight of the stem	0.69	0.10	0.07	*	0.56	0.08	0.06	*	0.62	0.09	0.07
Average number of leaves of the stem	0.33	0.05	0.04	*	0.58	0.13	0.09	**** *	0.46	0.09	0.07
Overall average	0.60	0.15	0.11		0.67	0.12	0.09		0.64	0.14	0.10
Correlation (r) between Q_ST_B_ and h^2^_*B*_	0.499	0.504				0.187				0.374	
Significance level of the correlation (P)	0.118	*P* > 0.05				*P* > 0.05				*P* > 0.05	

*P < 0.05; ***P < 0.001; ****P < 0.0001; *****P < 10^–5^; n.s., not significant (Tukey-Kramer multiple comparison test). As for all of the traits, the differences among plants within groups were always highly significant (P < 10^–5^); for brevity, only the significance levels for the between-groups source of variation are given. For each trait, the value of the Q_ST_B_ is reported; Q_ST_B_ values are reported assuming F_IS_ = 0.00, or for the putatively neutral F_IS_ = −0.315 estimated from the SSR data. The results of the F_ST_ - Q_ST_ comparisons are reported: underlined Q_ST_ values exceeded the upper 95% limit of the putatively neutral F_ST_ among the SSR groups; Q_ST_ values in bold exceeded the upper 99.5% limit; Q_ST_ values in bold and underlined exceeded the upper 99.9% limit.

### Relationship Between Molecular and Quantitative Trait Variation

At the within-group level, the correlation between H_E_ and the average h^2^_B_ was positive but not significant (Pearson *r* = 0.72; *n* = 5; *p* > 0.05).

**FIGURE 3:**
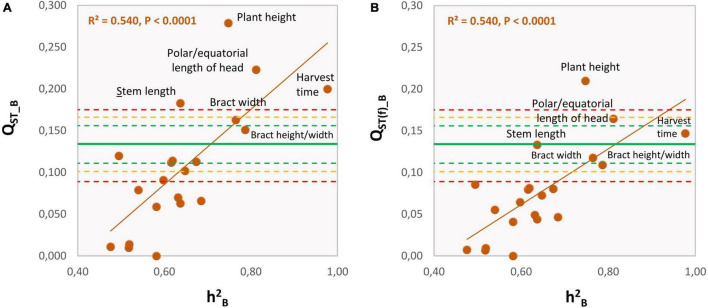
Relationships between the level of quantitative-genetic subdivision among the SSR groups (Q_ST_B_) and the average heritability within the SSR groups (h^2^_B_) for the 21 phenotypic traits recorded at the harvest of the first head, as listed in Table 3. Q_ST_B_ was calculated assuming F_IS_ = 0.00 **(A)** or for the neutral F_IS_ = –0.315 estimated from the SSR data **(B)**. Orange continuous line, regression line; green continuous line: average level of genetic subdivision among groups (F_ST_) based on putatively neutral SSRs; green, yellow, and red dashed lines: lower and upper limits (95%, 99%, 99.9%, respectively) of the neutral F_ST_ based on bootstrap.

There was a strong and positive correlation between Q_ST_B_ and the average within-groups h^2^_B_ (REML method: *r* = 0.736, *n* = 21, *p* = 0.0001; 95% C.I.: 0.446–0.887; Figure 3A). Furthermore, of the 21 quantitative traits measured at the harvest of the first head, four had Q_ST_B_ values that exceeded the C.I. for the molecular, putatively neutral F_ST_ (Figure 3A and Table 3). These were plant height, head shape (as polar/equatorial head-length ratio), harvest time, and stem length, which exceeded the upper C.I._99.9%_. Furthermore, the bract width was also significant as it was outside the C.I._95%._ Among these traits, head shape, stem length and, to a lesser extent, harvest time, confirmed their outlying behaviors also at the stages of the secondary and tertiary heads (Table 4). For the “total weight of heads,” Q_ST_ > F_ST_ for the secondary heads, but not for the tertiary heads. The correlation between the degree of quantitative-genetic subdivision among populations (Q_ST_) and the average heritability within populations (h^2^_B_) did not reach statistical significance (*p* = 0.118, 0.573, respectively). Based in Q_ST(f)_B_, the traits “plant height” and “head shape” remain statistically significant (Figure 3B) exceeding the C.I._95%_ and C.I._99.9%_ for the neutral F_ST_.

**FIGURE 4:**
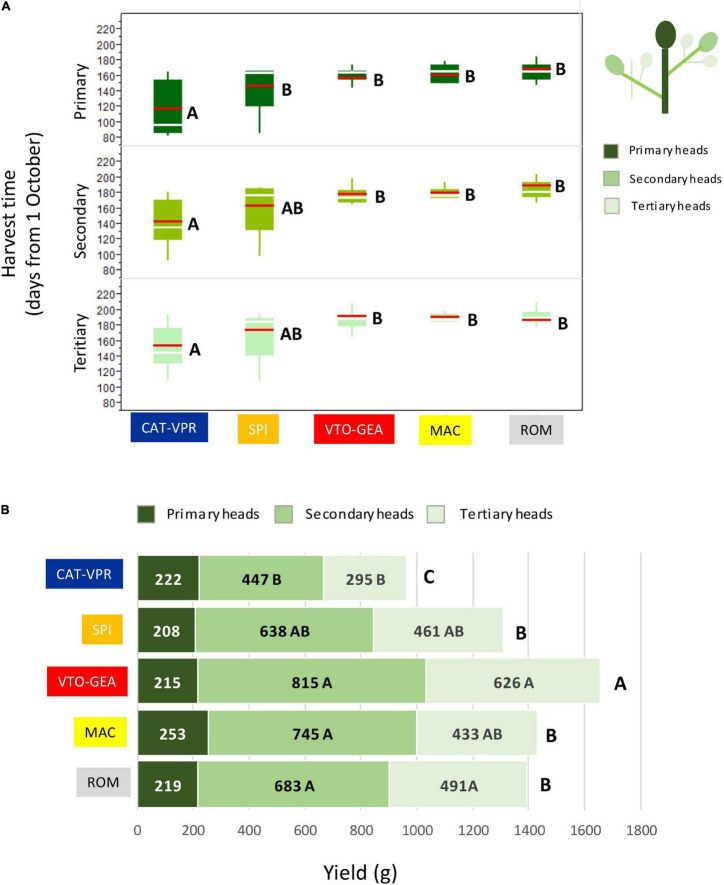
Differences between the SSR groups for the average harvest time **(A)** and yields of primary, secondary, and tertiary heads **(B)**. Group means are compared referring to the same head type (i.e., primary, secondary, and tertiary) and means with different letters are significantly different (*p* < 0.05; Tukey-Kramer HD test). In panel **(A)** green boxes = interquartile range; red bar = mean; white bar = median.

The highest level of population structure that separated CAT_VPR from the other groups (*K* = 2; Figure 1) was associated with an abrupt variation in the harvest time (Figure 4A). Indeed, the CAT-VPR group was 35–42 days earlier, on average, than the other groups. The SPI group, which split at *K* = 3 (Figure 1), tended to be the earliest among the late groups, for both the secondary and tertiary heads (Figure 4A). Furthermore, the differences between the SSR-derived groups for yield performance paralleled those for harvest time (Figure 4B). Indeed, the correlation between these two variables was positive and significant (REML: *r* = 0.940; C.I._95%_ = 0.340–0.997; *p* = 0.0174), i.e., the yield increased with the lateness. Thus, the CAT-VPR yield was 41.7% lower than that of VTO-GEA, the most productive group (Figure 4B). The ROM, MAC, and SPI groups tended to have intermediate positions. In all of the groups, the secondary heads accounted for ∼50% of the total yield (from 46% to 52%). The variation between groups for the yield components was also notable (Table 5). This was particularly evident for the number of secondary and tertiary heads (Table 5), where the top-ranking group was VTO-GEA (4.1 and 4.8, respectively), while the bottom-ranking group was CAT-VPR (2.9 and 2.6, respectively). The CAT-VPR group also occupied the last position for the weights of the heads (157 g and 113 g, respectively), while the MAC group tended to have larger heads, particularly for the primary and secondary heads (253 and 209 g; Figure 4B and Table 5, respectively).

**TABLE 5:** Average numbers and weights of the secondary and tertiary heads for the five SSR-derived groups of cultivated globe artichokes.

Group	Number (n)		Weight (g)	
	Secondary	Tertiary	Secondary	Tertiary
CAT-VPR	2.9 a	2.6 a	157 a	113 a
ROM	3.7 ab	3.7 b	181 ab	125 b
MAC	3.4 ab	3.6 b	209 b	123 b
SPI	3.2 a	3.5 b	196 b	123 b
VTO-GEA	4.1 b	4.8 c	203 b	129 b
Range (%)	41.3	84.6	29.3	14.1

*Data are means, where those with different letters are significantly different (p < 0.05; Tukey’s honestly significant difference multiple comparisons test).*

**FIGURE 5:**
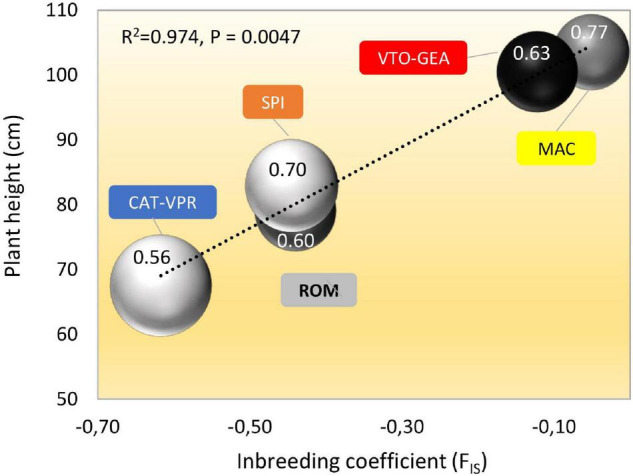
Relationships between coefficient of inbreeding (F_IS_), plant height (cm), gene diversity (H_E_), linkage disequilibrium (LD), and average heritability (h^2^_B_) for the five SSR groups of globe artichokes. Bubble size is proportional to the level of LD within the groups (as% of pair of loci with *p* < 0.05; Table 2); bubble shading (darkness) is proportional to the level of H_E_ (Table 2). The number within each bubble indicates the average h^2^_B_ across the quantitative traits.

Bract shape (as width and height/width ratio) varied according to clustering history (Table 6). Indeed, CAT-VPR showed the most oblong bracts, while VTO-GEA, MAC, and ROM showed the narrowest and most rounded bracts. The SPI group occupied a transitional position. For head shape (as polar/equatorial length ratio), the SPI showed the most oblong head (Table 6). Two close groups based on SSRs, MAC, and ROM, both showed the most spherical heads while VTO and CAT showed intermediate characteristics. Plant height and stem length did not follow the history of clustering based on Structure (Table 6). The average plant height was positively correlated with the average inbreeding coefficient, F_IS_ (Figure 5). As the group-specific F_IS_s were all negative, this meant that the lower the heterozygote excess, the taller the plants. Furthermore, when the plant stature increased, H_E_ and h^2^_B_ also increased, while LD tended to decrease (Figure 5).

**TABLE 6:** Differences between SSR-derived groups for plant height, stem length, and head and bract shape.

SSR-derived group	Plant height (cm)	Stem length (cm)	Head shape (*p*/E)	Bract shape	
				Width (mm)	Height/width
CAT-VPR	67.5 b	28.6 ab	1.17 ab	42.7 a	0.43 b
SPI	82.9 ab	29.8 ab	1.2 ab	41.6 ab	0.46 ab
VTO-GEA	100.5 a	29.7 ab	1.19 ab	38.3 b	0.48 a
MAC	103.5 a	36.1 a	1.06 bc	38.0 ab	0.49 a
ROM	79.1 b	22.6 b	1.01 c	35.3 b	0.49 a

*p/E, polar/equatorial length ratio.*

*Data are means, where those with different letters are significantly different (p < 0.05; Tukey’s honestly significant difference multiple comparisons test).*

### Comparison Between Molecular and Qualitative Trait Variations

**FIGURE 6:**
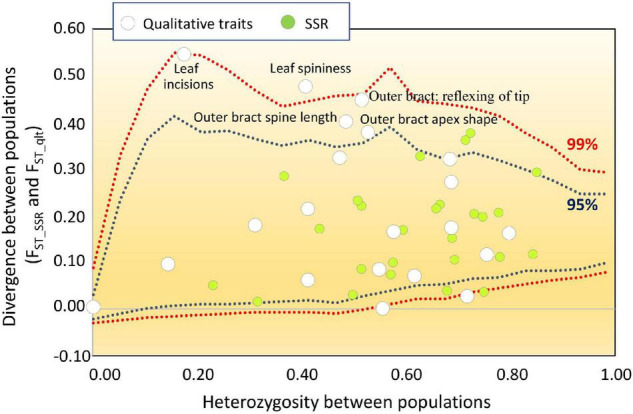
F_ST_ outlier analysis for qualitative traits. White dots, qualitative traits; green dots, SSR loci. The qualitative traits were treated as multiallelic loci. The divergence between populations was calculated for both qualitative trait (F_ST_qlt_) and SSR (F_ST_SSR_) conditioned to the heterozygosity between populations. The data obtained for the qualitative traits were then overlapped with the neutral envelope obtained by simulation using 26 SSRs. Blue and red dotted lines, 95% and 99% limits of the neutral envelope, respectively.

Among the 23 UPOV descriptors applied, 20 were polymorphic throughout the entire collection (Table 7). Within each group, the number of polymorphic traits ranged from 15 for ROM and SPI, to 20 for VTO-GEA (Table 7). VTO-GEA was the group with the highest diversity, based on both the number of phenotypic classes, n_c_, (2.73) and Nei’s diversity index, I_Nei_, (0.43), while the lowest I_Nei_ (0.28) was recorded for CAT-VPR (Table 7). This confirmed the observation based on the average h^2^_B_ within the groups and the average H_E_ with SSRs. The average divergence for qualitative traits (F_ST_qlt_) among the five SSR-derived groups was 0.20. This is close to the estimated value for the SSR markers (F_ST_ = 0.17) and quantitative traits (Q_ST_B_ = 0.18). However, F_ST_qlt_ varied widely among the traits (Figure 6). In five cases, F_ST_qlt_ was large enough to fit outside the “neutral envelope” obtained by simulation using SSRs (Figure 6). The best evidence here was for leaf incision (F_ST_qlt_ = 0.54; *p* < 0.01) and leaf spininess (F_ST_qlt_ = 0.8, *p* = 0.01), followed by bract curvature (F_ST_ql t_ = 0.44, *p* < 0.05), spine length (F_ST_qlt_ = 0.40, *p* < 0.05) and apex shape (F_ST_qlt_ = 0.38, *p* < 0.05).

**TABLE 7:** Diversity statistics (Nei’ diversity index, I_Nei_, and number of phenotypic classes, n_c_) for the 23 UPOV descriptors applied within and between the five SSR groups of the cultivated globe artichoke.

Trait	Particular	CAT-VPR		ROM		MAC		SPI		VTO-GEA		n_*c*_			Nei diversity index (I_*Nei*_)		
		n_*c*_	I_*Nei*_	n_*c*_	I_*Nei*_	n_*c*_	I_*Nei*_	n_*c*_	I_*Nei*_	n_*c*_	I_*Nei*_	Average	s.d.	Total	Average	s.d.	Total
Lobe	Shape of tip (excluding terminal lobe)	1	0.00	1	0.00	1	0.00	1	0.00	1	0.00	1.0	0.00	1	0.00	0.00	0.00
	Shape of tip of secondary lobes	1	0.00	1	0.00	1	0.00	1	0.00	1	0.00	1.0	0.00	1	0.00	0.00	0.00
Leaf	Attitude	2	0.11	2	0.33	2	0.39	1	0.00	3	0.57	2.0	0.71	3	0.28	0.23	0.43
	Spininess	1	0.00	1	0.00	2	0.20	3	0.60	2	0.06	1.8	0.84	3	0.17	0.25	0.14
	Incisions	3	0.53	1	0.00	1	0.00	1	0.00	2	0.06	1.6	0.89	3	0.12	0.23	0.36
	Hairiness on upper side	1	0.00	1	0.00	1	0.00	1	0.00	1	0.00	1.0	0.00	1	0.00	0.00	0.00
Leaf blade	Intensity of green color (upper side)	2	0.51	3	0.69	2	0.39	2	0.33	3	0.68	2.4	0.55	3	0.52	0.16	0.66
	Hue of green color	3	0.41	1	0.00	2	0.39	2	0.33	2	0.50	2.0	0.71	3	0.33	0.19	0.41
	Intensity of gray hue	2	0.51	2	0.51	3	0.67	2	0.60	3	0.57	2.4	0.55	3	0.57	0.07	0.55
	Blistering	3	0.38	3	0.65	3	0.60	3	0.73	4	0.51	3.2	0.45	4	0.58	0.13	0.53
Petiole	Anthocyanin coloration at base	4	0.69	4	0.75	2	0.56	2	0.60	4	0.75	3.2	1.10	4	0.67	0.09	0.71
Central flower head	Shape in longitudinal section	3	0.54	4	0.75	4	0.78	3	0.73	5	0.74	3.8	0.84	5	0.71	0.09	0.73
	Shape of tip	2	0.36	3	0.64	3	0.73	3	0.73	3	0.39	2.8	0.45	4	0.57	0.18	0.62
	Time of appearance	5	0.72	3	0.64	4	0.73	3	0.73	4	0.60	3.8	0.84	5	0.68	0.06	0.76
	Anthocyanin coloration of inner bracts	3	0.42	2	0.51	2	0.36	2	0.60	3	0.55	2.4	0.55	3	0.49	0.10	0.55
	Density of inner bracts	3	0.46	2	0.51	3	0.69	3	0.80	3	0.64	2.8	0.45	3	0.62	0.14	0.60
Outer bract	Main shape	1	0.00	2	0.44	2	0.20	1	0.00	3	0.17	1.8	0.84	3	0.16	0.18	0.14
	Apex shape	2	0.16	1	0.00	3	0.38	2	0.33	3	0.59	2.2	0.84	3	0.29	0.22	0.48
	Color (external side)	3	0.26	3	0.56	3	0.64	2	0.33	4	0.72	3.0	0.71	4	0.50	0.20	0.64
	Reflexing (of tip)	3	0.11	2	0.44	2	0.20	1	0.00	3	0.49	2.2	0.84	3	0.25	0.21	0.45
	Spine length	2	0.11	1	0.00	2	0.20	3	0.60	3	0.54	2.2	0.84	5	0.29	0.27	0.43
	Mucron	1	0.00	2	0.51	2	0.36	1	0.00	2	0.45	1.6	0.55	2	0.26	0.25	0.29
Receptacle	Shape in longitudinal section	2	0.21	3	0.56	2	0.56	2	0.33	2	0.30	2.2	0.45	3	0.39	0.16	0.38
	**Mean**	**2.30**	**0.28**	**2.09**	**0.37**	**2.26**	**0.39**	**1.96**	**0.37**	**2.78**	**0.43**	**2.28**	**0.314**	**3.13**	**0.37**	**0.05**	**0.49**
	**s.d.**	**1.06**	**0.24**	**1.00**	**0.29**	**0.86**	**0.26**	**0.83**	**0.31**	**1.04**	**0.26**	**0.96**	**0.108**	**1.14**	**0.27**	**0.03**	**0.18**

**FIGURE 7:**
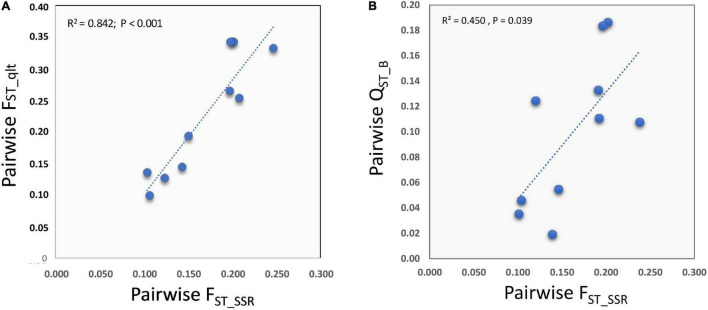
Relationships between the degree of phenotypic divergence for qualitative **(A)** and quantitative **(B)** traits for the phenotypic traits listed in Supplementary Table 3 and the pairwise genetic distances between groups for the SSR markers.

Interestingly, there was strong correlation between the qualitative traits and SSR distances between the pairs of groups (REML: *r* = 0.918; *n* = 10; L_95%_ = 0.685; U_95%_ = 0.981; *p* = 1.76 × 10^–4^) (Figure 7A). This was stronger than the positive and marginally significant correlation observed between quantitative-genetic divergence (Q_ST_B_) and SSR genetic divergence (F_ST_) between the pairs of SSR-derived groups (REML *r* = 0.670, *n* = 10; L_95%_ = 0.070, U_95%_ = 0.914; *p* = 0.034; Spearman-rank ρ = 0.636; *p* = 0.048) (Figure 7).

## Discussion

### The Globe Artichoke Cultivated Gene Pool Clusters Into Five Subgroups

This study shows that the analyzed globe artichoke accessions clustered into two main genetic groups. The first comprises the Catanesi and Violetti di Provenza types and is quite uniform. The second is more complex and includes four additional subgroups related to Spinosi, Green et al., Violetti di Toscana, Romaneschi, and Macau.

Based on the phenotypic traits and multivariate analysis, Elia and Miccolis (1996) distinguished five main morphophenological clusters: “early,” “medium-early,” “late with small head,” “late-violet,” and “late with large head.” This genetic classification based on SSRs shows good agreement with that of Elia and Miccolis (1996) based on the phenotypic data. Indeed, their “early” and “medium-early” clusters correspond to our CAT-VPR group, which is composed of Catanesi and Violetti di Provenza, and is characterized by long-shaped and small main and secondary heads, low-medium yield, early harvest, and plant with low vigour (short, with low numbers of heads). Their “late with small head” cluster corresponds to our SPI group, which includes Spinosi types characterized by long-shaped and medium-small heads and late harvest. The “late-violet” cluster corresponds to VTO-GEA that includes “Violetti di Toscana” and several other varieties assigned to the category “Green et al.”. On average, these plants were vigorous (tall and high-yielding) and showed less elongated heads than the previous groups. Finally, the “late with large head” cluster correlates with our ROM and MAC groups, which are characterized by lateness and large sizes of the principal and secondary heads, with a shape that tends to be spherical.

Based on AFLP markers, Lanteri et al. (2004) reported that Catanesi and Violetti di Provenza were closer to Romaneschi and Macau than to Spinosi and Violetti di Toscana. Differently, in our study, Romaneschi and Macau are closer to Violetti di Toscana and to several varieties classified as “Green et al.”. As the collections analysed in Lanteri et al. (2004) and in our study are similar, the observed differences could be inherent to the different markers used (AFLP vs. SSR). Indeed, AFLPs, are biallelic and dominant markers, while SSRs are multiallelic and codominant markers. Moreover, AFLPs and SSR markers have different mutational models and rates. The mutation rates of SSRs are between 10^–3^ and 10^–4^, as inferred from genetic data (Estoup and Angers, 1998; Mariette et al., 2001; Garoia et al., 2007) and estimated from observed mutations (Vigouroux et al., 2002; Thuillet et al., 2005). The mutation rate for AFLPs is lower being between 10^–6^ and 10^–5^ (Mariette et al., 2001; Gaudeul et al., 2004; Kropf et al., 2009). On this basis, different marker types can lead to different precision when assessing relationships between single individuals, as well as to different estimations of within- and between-population components of genetic diversity (see, e.g., Gaudeul et al., 2004). Moreover, high evolutionary rates necessarily result in low phylogenetic signals, and conversely, low evolutionary rates result in high phylogenetic signals (Revell et al., 2008); thus, with their slower mutation rate, AFLP markers can highlight more ancient population splits (Flanders et al., 2009). Therefore, the population genetic structure described by Lanteri et al. (2004) might represent a more ancient one than that depicted in this study.

### The Signature of Long-Term Clonal Propagation

The chloroplast and nuclear genetic polymorphisms as well as the molecular and phenotypic population structures, were associated. This is expected if there are multiple germplasm sources contemporarily differentiated for nuclear, organellar loci, and phenotypic traits. Furthermore, LD was detected within each of the five groups, and the diversity statistics based on SSRs and quantitative and qualitative traits are correlated. These results indicate that the five groups of globe artichoke varieties were genetically relatively isolated, and that the recombination accumulated through crop evolution was overall not sufficiently high (or the time elapsed was too low) to break up the cytonuclear and marker-trait LD. The presence of LD in the whole dataset is also indicated by the correlation between the divergence of the group for SSR markers (F_ST_) and the divergence for the quantitative traits (Q_ST_B_) and the qualitative traits (F_ST_qlt_).

Within the groups, in addition to LD, we observed negative F_IS_, which indicates an excess of heterozygotes compared to the Hardy-Weinberg expectation. Detecting LD is not sufficient to call for clonal reproduction (de Meeûs and Balloux, 2004; Halkett et al., 2005). However, negative F_IS_ is the ultimate signature of clonal diploid populations (Halkett et al., 2005). These results are consistent with the expected structure of genetic diversity of a collection of artichoke cultivars where clonal propagation is a common practice; in this species, propagation is carried out with “*carducci*” (basal shoots) or “*ovoli”* (semi-dormant shoots with a limited root system). Furthermore, our study also shows that some groups have a higher excess of heterozygotes than others and this suggests that different breeding histories and various degrees of historical “clonality” characterize the evolution of the five identified genetic groups.

Albeit selfing is not precluded, cross-fertilization in globe artichoke is promoted by protandry (Mauromicale and Ierna, 2000). In a recent re-sequencing study Acquadro et al. (2017) documented the significant hetorozigosity of some cultivated clonal varieties. Consistent with this finding, Basnitzki and Zohary (1987) showed that the (self) seed progenies of clonal varieties showed wide segregation of morphological and production traits. Thus, it can be hypothesized that the excess of heterozygotes at SSR loci might reflect the selection of plants with superior hybrid vigor because of the heterozygote advantage (overdominance). However, genomic surveys have suggested that loci with heterozygote advantage must be considered only a small minority of all loci in a species (Hedrick, 2012). Furthermore, complete clonality is comparable to a long-term population bottleneck with a population size of one, which implies that heterozygote excess is also expected if the selection is not acting (Balloux et al., 2003). We also observed that the groups with a strong excess of heterozygotes (and strong LD, low H_E_, low h^2^_B_) were shorter and produced fewer and smaller heads (i.e., were less vigorous) than groups with low heterozygotes (and lower LD, higher H_E_, higher h^2^_B_). This suggests that selection against deleterious mutations might be less effective in groups with high clonality (and high heterozygote excess) than in those with low clonality (with low or null heterozygote excess). Indeed, when there is no recombination, selection against deleterious mutations is less effective, and deleterious mutations have the potential to accumulate (i.e., the mutation load) (McKey et al., 2010; Gaut et al., 2015). The accumulation of mutations in a purely clonal line ultimately leads to lower fitness, which is associated with lower agronomic performance (McKey et al., 2010). However, in globe artichoke, this remains to be demonstrated with a dedicated study.

### Comparison Between Molecular and Phenotypic Population Structures Indicates a Role for Divergent Selection

Under neutral evolution (i.e., absence of selection), Q_ST_ = F_ST_ is expected, such that the proportion of total variance allocated among populations (or genetic groups, in this case) should be equal or similar for both quantitative traits and molecular markers (Leinonen et al., 2013). Therefore, assuming that F_ST_ has been estimated considering putatively “neutral” SSR loci, the inequality Q_ST_ > F_ST_ indicates divergent selection among the populations. Indeed, this suggests that for a given quantitative trait, populations (or genetic groups) show different mean values not only because of random demographic processes (e.g., genetic drift, migration, mutation), but also due to the selection that exerts differential pressure among the populations. On the contrary, the inequality Q_ST_ < F_ST_ suggests uniform selection across populations: populations tend to have the same trait means because the selection has the same direction in all of the populations.

Following this rationale, we compared the F_ST_ and Q_ST_B_ obtained among the genetic groups of the globe artichokes identified by the analysis of the population structure. This comparison suggests that some quantitative traits have an “excess” of divergence between groups (Q_ST_B_) compared to the putatively neutral markers (F_ST_) (Q_ST_B_ > F_ST_). For these traits, divergent selection, rather than demographic factors, might be responsible for the phenotypic differences observed between the genetic groups. These traits are harvest time, plant height, head shape, and stem length. Among the qualitative traits, divergent selection can be suggested for leaf spininess, spine length, apex shape, and reflexing of the tip of the outer bracts as they showed an F_ST__qlt that excedeed the “neutral” F_ST_. In general, it is possible that these traits were under the farmer’s attention when selecting plants for clonal propagation, as they strongly condition both agronomic practice (e.g., harvest time) and consumer preference (e.g., stem length, head shape, spininess). However, caution should be exercised when interpreting these data. Indeed, deviations from the Hardy-Weinberg equilibrium might affect the Q_ST_–F_ST_ comparison. Inbreeding (F_IS_ > 0) can lead to Q_ST_ < F_ST_ under neutrality (instead of Q_ST_ = F_ST_), which results in low power when detecting divergent selections. On the contrary, when, as in the present case, there is an excess of heterozygotes (F_IS_ < 0), it is possible that under neutrality Q_ST_ > F_ST_ (instead of Q_ST_ = F_ST_), which leads to a higher risk of false positives (Cubry et al., 2017). As expected, when the Q_ST_ is calculated considering our negative neutral F_IS_ estimate, the Q_ST_ values are reduced. However, the traits “plant height” and “head shape” remain statistically significant.

For the trait “plant height,” Q_ST_ > F_ST_ is often observed in both trees (Yang et al., 1996) and herbaceous plants (Waldmann and Andersson, 1998; Kawakami et al., 2011; Eriksen et al., 2012; Michalski et al., 2017). Interestingly, very high Q_ST_s were observed for plant height, flowering date, and capitulum size (diameter) in the sunflower species *Helianthus maximilian*, which like *C. cardunculus var. scolymus* belongs to the Asteraceae family, is perennial and is diploid (Kawakami et al., 2011). Variations in these traits are relevant to local adaptation to spatially heterogeneous environments. Furthermore, as might also be suggested for our study, Kawakami et al. (2011) noted that plant height and flowering time might have both been found under divergent selection because of the typical life-history trade-off between growth and reproductive timing (Obeso, 2002). The existence of this trade-off in cultivated artichoke was suggested by Crinò et al. (2008). Alternatively, plant height and flowering time might at least partially share the same genetic control, as has been suggested in other plant species (Tondelli et al., 2014; Bustos-Korts et al., 2019).

The Q_ST_–F_ST_ comparison assumes that the genetic control of the quantitative traits is additive (Spitze, 1993). Non-additive (dominance or epistasis) and maternal effects might have different weights for different traits and might confound any inference (Leinonen et al., 2013). It is possible that traits with simple genetic control, a strong dominance variance component, and under certain demographic models show Q_ST_ inflated over the neutral expectation, mimicking divergent selection (López-Fanjul et al., 2003, 2007). However, such inflation is unlikely for traits with polygenic control (Goudet and Martin, 2007), and, in general, with non-additive inheritance, the Q_ST_–F_ST_ approach to look for diversifying selection would be conservative (Whitlock, 1999, 2008; Cubry et al., 2017). Furthermore, the Q_ST_–F_ST_ approach can be suggested as an exploratory tool (Leinonen et al., 2008; Whitlock, 2008), particularly when detailed information on phenotypic traits is not available. In this case, as Whitlock (2008) noted, “the difficult statistical properties of Q_ST_ become less important, and if the Q_ST_ of several traits is measured, then the traits with the highest Q_ST_ values may be good candidates for further study of selection.” In other words, non-additive and maternal effects probably did not significantly alter the conclusions of this study, and plant height and head shape followed by harvest time, stem length, and the number of heads are good candidates for having been subjected to divergent selection during globe artichoke evolution.

Another observation indicates a role for divergent selection among the genetic groups. Indeed, we found that although there is a substantial amount of variation among the traits for Q_ST_B_, much of this variation appears to be associated with variation in h^2^_B_. Under neutrality, Q_ST_ does not correlate with h^2^_B_ (Lande, 1992). In contrast, the response to directional selection on a quantitative trait (and hence the Q_ST_B_ between populations), *ceteris paribus*, is proportional to h^2^_B_ (Falconer and Mackay, 1996). Therefore, the positive correlation between Q_ST_ and h^2^_B_ observed in this study is qualitatively consistent with the hypothesis that selection contributed to the phenotypic differences observed between the groups. In previous studies on *Pinus contorta* (Yang et al., 1996), *Medicago truncatula* (Bonnin et al., 1996), and *Clarkia dudleyana* (Podolsky and Holtsford, 1995), the correlation between Q_ST_ and h^2^_B_ was also positive; a similar situation has also been observed in animals, such as in the microcrustacean *Daphnia pulex* (Lynch et al., 1999).

## Conclusion

This study shows that a globe artichoke collection that comprises most of the varieties cultivated worldwide has a clonal population structure and is moderately structured. The data consistently show that five genetic groups with different degrees of “historical” clonality characterize the domesticated gene pool of the globe artichoke. Clonal propagation was probably accompanied by clonal selection for a set of different phenotypic characteristics. The traits that affect phenology (harvest time), plant architecture (plant height, stem length), head shape, and spininess (of both leaves and bracts) are suggested as the main targets of selection. The overall covariance between the molecular and phenotypic population structures indicates that genome-wide association mapping strategies must deal with the identified population structure to minimize the risk of false positives.

## Data Availability Statement

The original contributions presented in this study are included in the article/Supplementary Material, further inquiries can be directed to the corresponding author/s.

## Author Contributions

DR, GA, MR, and EP conceived and designed the experiments. LB, AP, and DS performed the field trial. LB, MR, and GA carried out field data curation. MR carried out DNA extraction. AA and CC performed SSR analysis. DR performed statistical analyzes. DR, EP, and CC wrote the manuscript. All authors contributed to the discussion, revised, and approved the final manuscript.

## Conflict of Interest

The authors declare that the research was conducted in the absence of any commercial or financial relationships that could be construed as a potential conflict of interest.

## Publisher’s Note

All claims expressed in this article are solely those of the authors and do not necessarily represent those of their affiliated organizations, or those of the publisher, the editors and the reviewers. Any product that may be evaluated in this article, or claim that may be made by its manufacturer, is not guaranteed or endorsed by the publisher.

## Footnotes

1
https://www.fao.org/faostat

## Supplementary Material

Supplementary Figure 1 Structure analysis. Evanno’s ΔK statistic as a function of the number “true” genetic populations assumed.

Supplementary Figure 2 Relationships among the varieties, as shown by the third and fourth discriminant linear functions. The former separated SPI and CAT-VPR from the other groups, and the latter mainly separated ROM and MAC.

Supplementary Figure 3 Individual to groups assignments by discriminant analysis of the principal components. Left: morphological types and name for each accession. Right: composition of each of the SSR genetic groups. Heat colors represent membership probabilities (red = 1, white = 0); blue crosses represent the prior cluster provided to DAPC. DAPC classification is consistent with the original clusters (blue crosses are on red rectangles).

Supplementary Figure 4 Variation at the chloroplast SSRs: **(A)** within the overall collection; **(B)** within and across the five nuclear SSR genetic groups of the globe artichoke varieties.
