# Supplementary material for: The Population Structure of a Globe Artichoke Worldwide Collection, as Revealed by Molecular and Phenotypic Analyzes
Source: Front Plant Sci. 2022 Jul 5;13:898740. doi: 10.3389/fpls.2022.898740 (PMC9294547; doi:10.3389/fpls.2022.898740)
Supplement: Supplementary file 1 [file Table_1.PDF]

Table S1. List of the 110 accessions in the living artichoke collection analyzed in this study. For each accession, the phenotypic typology is also provided.

N°	Variety/Clone	Phenotypic typology
**1**	Brindisino	**Catanese (CAT)**
**2**	Catanese	
**3**	Catanese	
**4**	Di Catania	
**5**	Di Niscemi	
**6**	Francesina	
**7**	Gagliardo Sgrò	
**8**	Locale di Cutrofiana	
**9**	Locale di Mola	
**10**	Locale di Ostuni	
**11**	Locale di Sibari	
**12**	Locale di Surbo	
**13**	Masedu	
**14**	Niscemese	
**15**	Romana	
**16**	Siracusano	
**17**	Violetto Precoce	
**18**	Camard	**Romanesco (ROM)**
**19**	Campagnano	
**20**	Castellamare	
**21**	Castellamare	
**22**	Centofoglie	
**23**	Di Vasto	
**24**	Grosso Orvietano	
**25**	Locale di Montelupo	
**26**	Locale di Strancona	
**27**	Mazzaferrata di Termoli	
**28**	Pasquaiolo	
**29**	Pietralcina	
**30**	Romanesco	
**31**	Romano	
**32**	Selezione Romanesco	
**33**	Terom	
**34**	Tonda di Paestum	
**35**	Di Palermo	**Spinoso (SPI)**
**36**	Spinoso di Gela	
**37**	Spinoso di Gonnos	
**38**	Spinoso di Palermo	
**39**	Spinoso di Sciacca	
**40**	Spinoso Sardo	
**41**	Spinoso violetto di Liguria	
**42**	110B/14 Gentile *	
**43**	110B/14 Selvatico *	
**44**	37/B Gentile *	
**45**	37/B Selvatico *	
**46**	Carloforte Guardiamori *	
**47**	Carloforte Puntanera *	
**48**	Meli Agris2 -C3 Gentile *	
**49**	Meli Agris2 -C3 Selvatico *	
**50**	Olmedo/Surigheddu *	
**51**	928	**Violetto di Provenza (VPR)**
**52**	932	
**53**	968	
**54**	AVM 7	
**55**	Baladi	
**56**	Banafsigi	
**57**	CP 11	
**58**	Hierois	
**59**	Violet d'Algerie	
**60**	Violet de Campagne	
**61**	Violet French	
**62**	Violetto di Gapeau	
**63**	Violetto di Margot	
**64**	Violetto di Provenza	
**65**	VP 45	
**66**	VP 49	
**67**	VP 556	
**68**	VP Massa	
**69**	Empolese	**Violetto di Toscana (VTO)**
**70**	Locale di Fano	
**71**	Moretto	
**72**	Nostrano violetto di Pesaro	
**73**	Precoce violetto di Chioggia	
**74**	Tema 2000	
**75**	Violetto	
**76**	Violetto di Maremma	
**77**	Violetto di S. Luca	
**78**	Violetto di Toscana	
**79**	Violetto di Toscana (II)	
**80**	Blanc Oranais	**Macau (MAC)**
**81**	Camus	
**82**	Caribou	
**83**	CB 641 (Camus Bretagne)	
**84**	CB 642 (Camus Bretagne)	
**85**	Gross Camus	
**86**	Macau	
**87**	Cacique	
**88**	A. Pigna	**Green *et al.* (GEA)**
**89**	Arruffara di Quartu	
**90**	Bayrampasa	
**91**	Bianco di Ostuni	
**92**	Bianco Tarantino	
**93**	Blanco	
**94**	Dash	
**95**	EB 9	
**96**	Emerald	
**97**	Green Globe	
**98**	Green Globe Thornless	
**99**	Imperial Star	
**100**	Kiss of Durgum	
**101**	Locale di Cuneo	
**102**	Locale di Parabita	
**103**	R 35	
**104**	Sakiz	
**105**	Selezione 67	
**106**	Testa di Ferro	
**107**	Tudela	
**108**	Verde di Pesaro	
**109**	Verde di Putignano	
**110**	Violetto di Putignano	

***** *'Spinoso sardo' clone selected by AGRIS***
