# Supplementary material for: The Population Structure of a Globe Artichoke Worldwide Collection, as Revealed by Molecular and Phenotypic Analyzes
Source: Front Plant Sci. 2022 Jul 5;13:898740. doi: 10.3389/fpls.2022.898740 (PMC9294547; doi:10.3389/fpls.2022.898740)
Supplement: Supplementary file 4 [file Table_4.PDF]

Table S4. Standardized differences test conducted with the Bottleneck software for the five SSR groups of the cultivated globe artichokes.

Single step mutation (%)	P-value for $H_E > H_{EQ}$ (i.e., presence of genetic bottleneck)				
	CAT-VPR	ROM	MAC	SPI	VTO-GEA
0	$<10^{-3}$	$<10^{-4}$	0.024	0.001	0.001
10	0.001	$<10^{-3}$	0.100	0.008	0.015
30	0.002	0.002	0.142	0.013	0.031
50	0.006	0.004	0.214	0.027	0.088
70	0.025	0.014	0.367	0.055	0.292
