# Supplementary figures and images for: The Population Structure of a Globe Artichoke Worldwide Collection, as Revealed by Molecular and Phenotypic Analyzes
Source: Front Plant Sci. 2022 Jul 5;13:898740. doi: 10.3389/fpls.2022.898740 (PMC9294547; doi:10.3389/fpls.2022.898740)

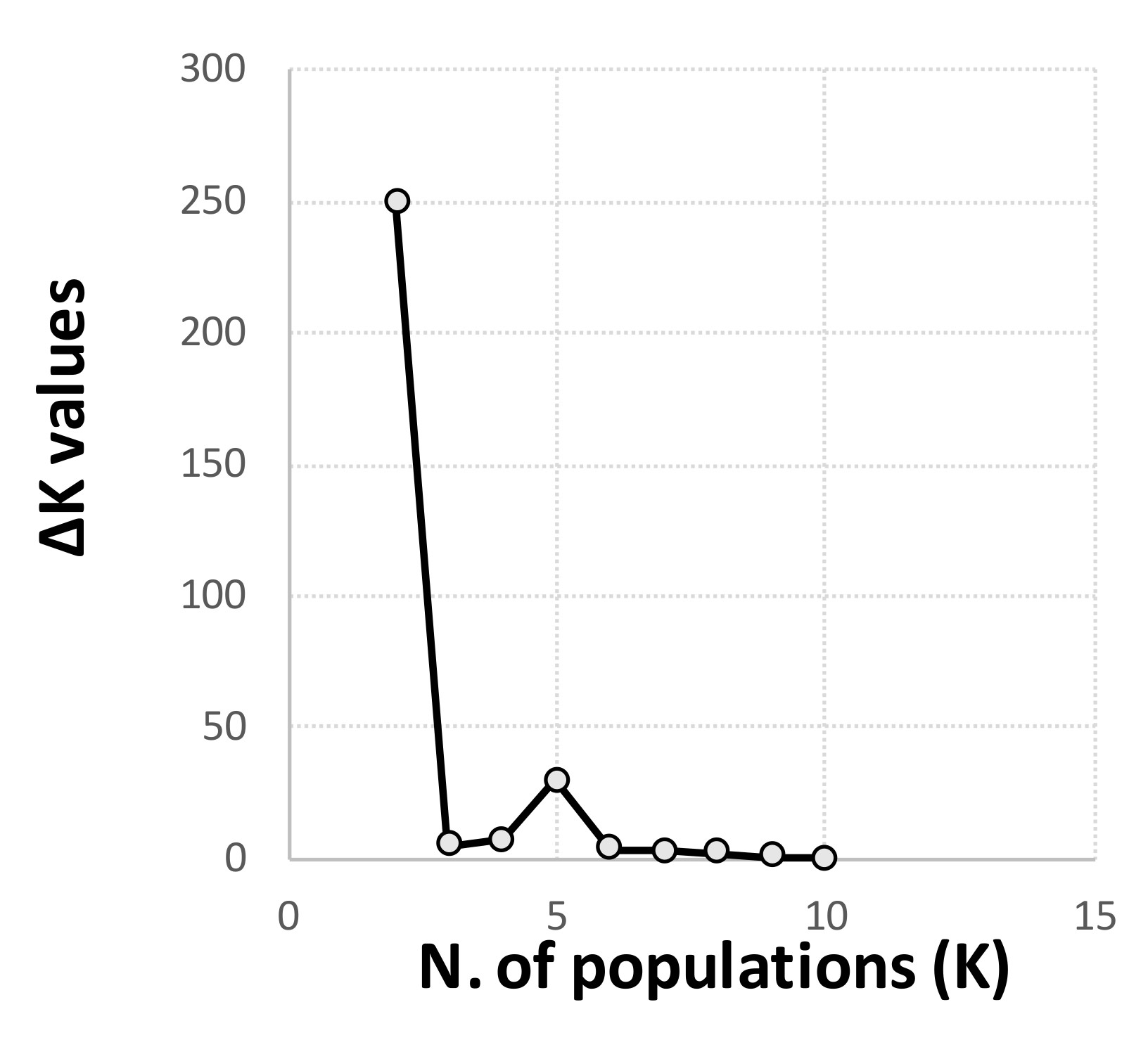

Supplement: Supplementary Figure 1 — Structure analysis. Evanno’s ΔK statistic as a function of the number “true” genetic populations assumed. [file Image_1.JPEG]

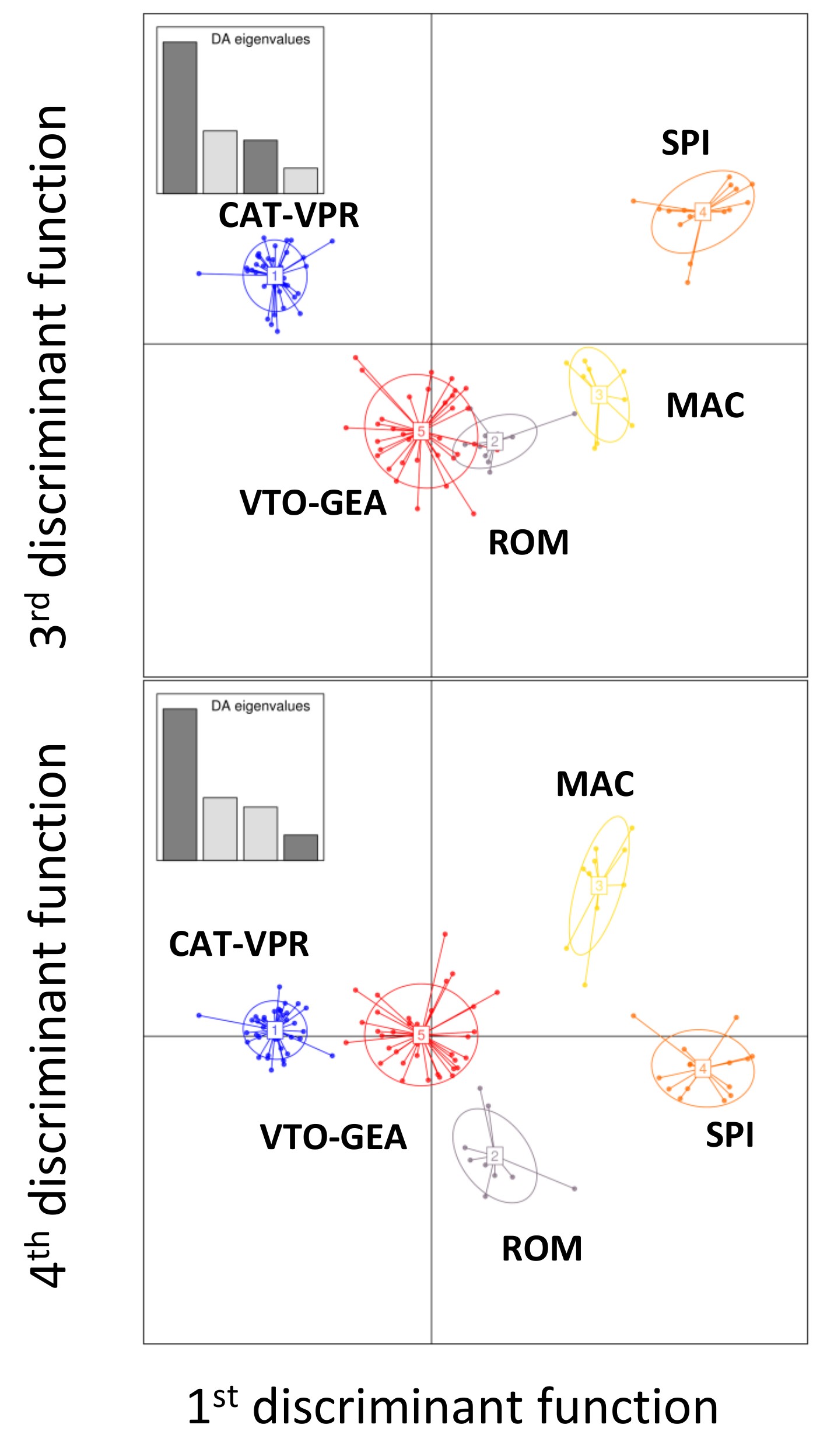

Supplement: Supplementary Figure 2 — Relationships among the varieties, as shown by the third and fourth discriminant linear functions. The former separated SPI and CAT-VPR from the other groups, and the latter mainly separated ROM and MAC. [file Image_2.JPEG]

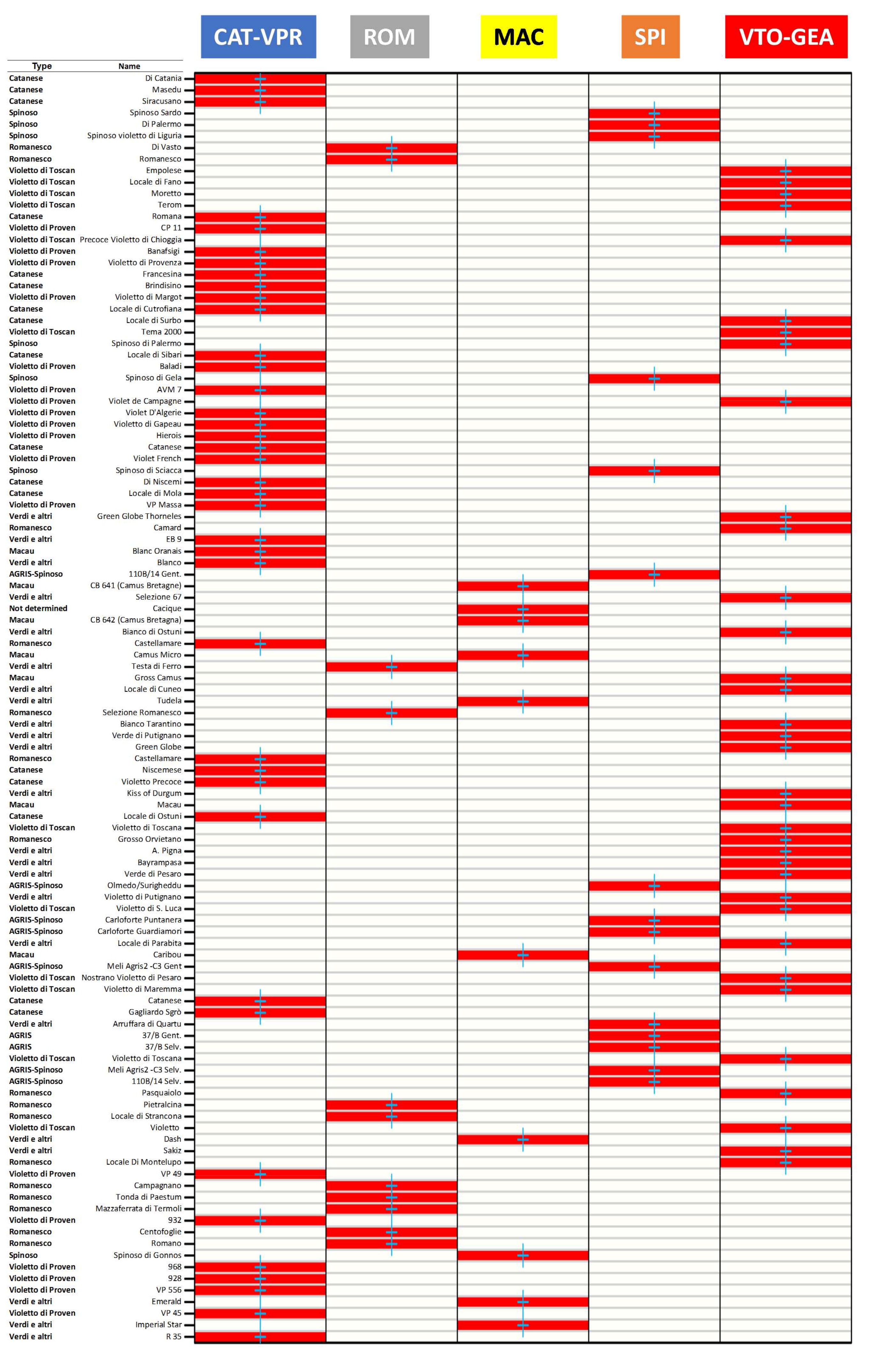

Supplement: Supplementary Figure 3 — Individual to groups assignments by discriminant analysis of the principal components. Left: morphological types and name for each accession. Right: composition of each of the SSR genetic groups. Heat colors represent membership probabilities (red = 1, white = 0); blue crosses represent the prior cluster provided to DAPC. DAPC classification is consistent with the original clusters (blue crosses are on red rectangles). [file Image_3.jpg]

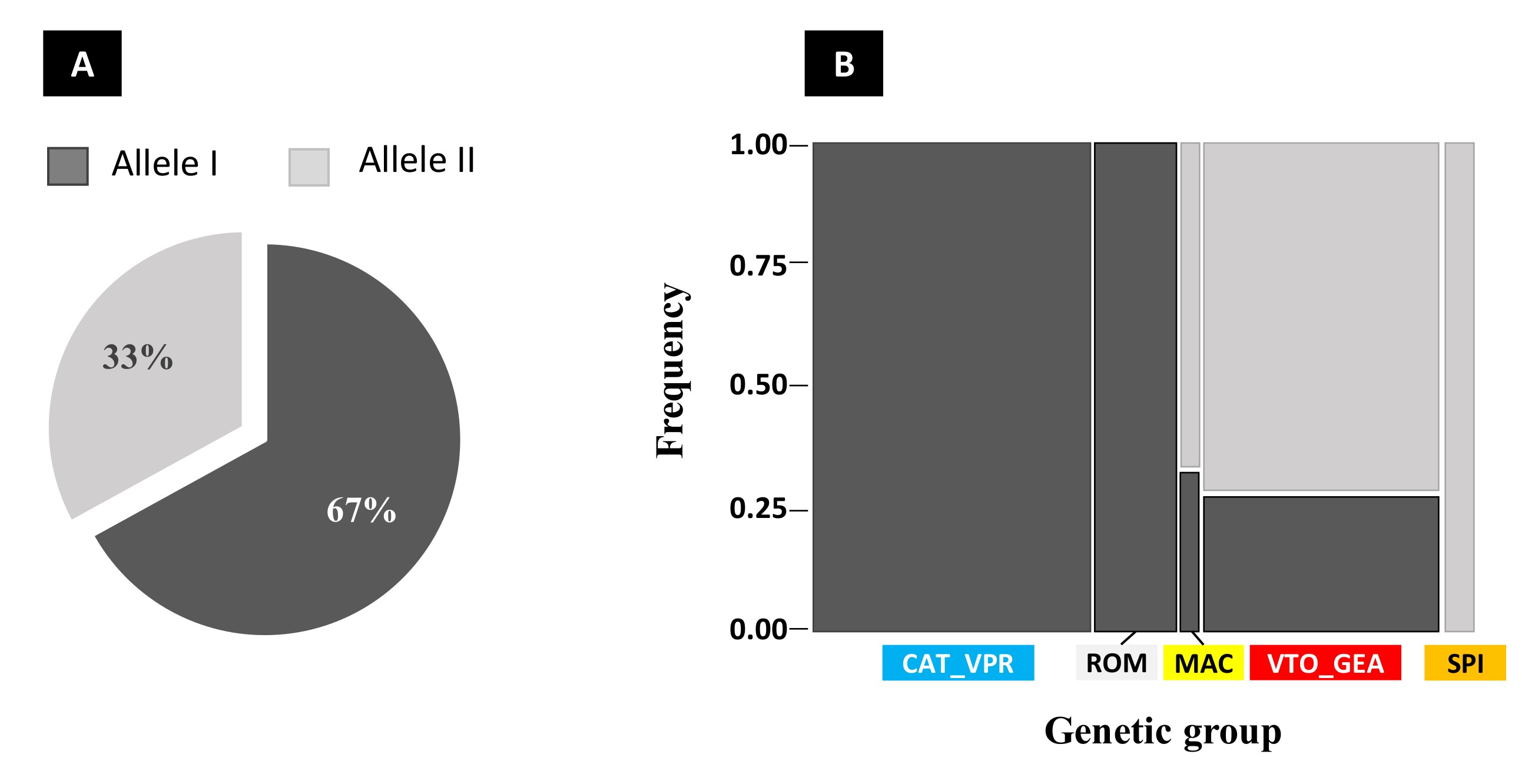

Supplement: Supplementary Figure 4 — Variation at the chloroplast SSRs: (A) within the overall collection; (B) within and across the five nuclear SSR genetic groups of the globe artichoke varieties. [file Image_4.JPEG]
